# Cluster energy prediction based on multiple strategy fusion whale optimization algorithm and light gradient boosting machine

**DOI:** 10.1186/s13065-024-01127-0

**Published:** 2024-01-30

**Authors:** Wu Wei, Li Mengshan, Wu Yan, Guan Lixin

**Affiliations:** 1 School of Physics and Electronic Information, Gannan Normal University, Ganzhou, 341000 Jiangxi China; 2 School of Mathematics and Computer Science, Gannan Normal University, Ganzhou, 341000 Jiangxi China

**Keywords:** Cluster, LightGBM, Energy prediction, Machine Learning

## Abstract

**Background:**

Clusters, a novel hierarchical material structure that emerges from atoms or molecules, possess unique reactivity and catalytic properties, crucial in catalysis, biomedicine, and optoelectronics. Predicting cluster energy provides insights into electronic structure, magnetism, and stability. However, the structure of clusters and their potential energy surface is exceptionally intricate. Searching for the global optimal structure (the lowest energy) among these isomers poses a significant challenge. Currently, modelling cluster energy predictions with traditional machine learning methods has several issues, including reliance on manual expertise, slow computation, heavy computational resource demands, and less efficient parameter tuning.

**Results:**

This paper introduces a predictive model for the energy of a gold cluster comprising twenty atoms (referred to as Au20 cluster). The model integrates the Multiple Strategy Fusion Whale Optimization Algorithm (MSFWOA) with the Light Gradient Boosting Machine (LightGBM), resulting in the MSFWOA-LightGBM model. This model employs the Coulomb matrix representation and eigenvalue solution methods for feature extraction. Additionally, it incorporates the Tent chaotic mapping, cosine convergence factor, and inertia weight updating strategy to optimize the Whale Optimization Algorithm (WOA), leading to the development of MSFWOA. Subsequently, MSFWOA is employed to optimize the parameters of LightGBM for supporting the energy prediction of Au20 cluster.

**Conclusions:**

The experimental results show that the most stable Au20 cluster structure is a regular tetrahedron with the lowest energy, displaying tight and uniform atom distribution, high geometric symmetry. Compared to other models, the MSFWOA-LightGBM model excels in accuracy and correlation, with MSE, RMSE, and R^2^ values of 0.897, 0.947, and 0.879, respectively. Additionally, the MSFWOA-LightGBM model possesses outstanding scalability, offering valuable insights for material design, energy storage, sensing technology, and biomedical imaging, with the potential to drive research and development in these areas.

**Graphical Abstract:**

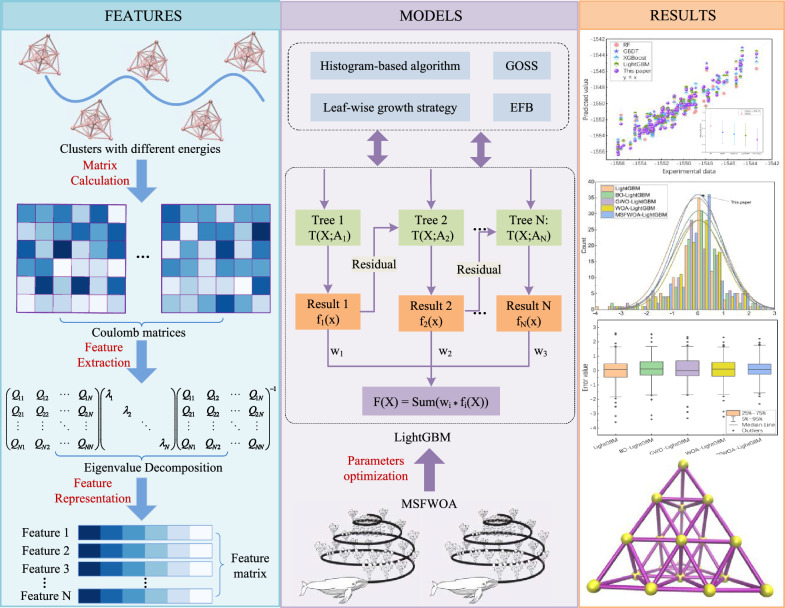

## Background

**Fig. 1:**
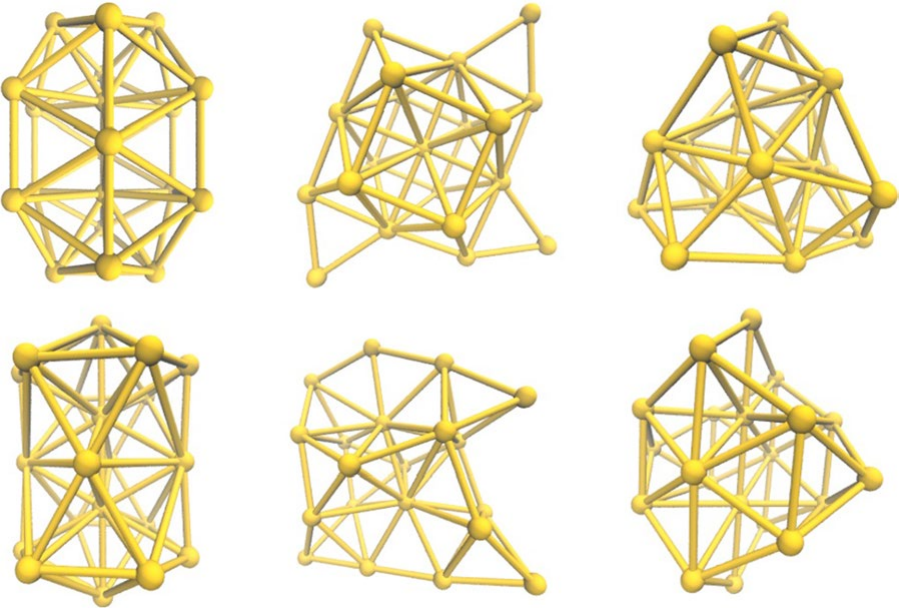
Six isomeric structures

Clusters represent a novel material structure and serve as an intermediate transition states during the transformation of substances from atoms and molecules to macroscopic objects. They are assemblies formed by the bonding of numerous atoms, molecules, or ions, driven by physical or chemical forces, with sizes ranging between one-tenth and a hundred nanometres [1–3]. Clusters [4–9], unlike individual atoms, molecules, and macroscopic solids, exhibit distinctive chemical reactivity and catalytic performance due to characteristics [10] such as quantum size effects, surface effects, and a higher surface area-to-volume ratio. Therefore, they find wide applications in fields including catalysis [11, 12], materials adsorption [13], biomedical applications [14], optic and optoelectronics [15–17]. The energy of clusters is pivotal for comprehending their stability and characteristics. Analysing and comparing these energies enhances our comprehension of the energy differences and relative levels among various clusters [18–20]. This in-depth understanding aids in predicting and explaining the electronic structure, magnetism, and optical properties of clusters. It leads to the optimization of the energy band structure of materials and the active sites of catalysts, and also enables advanced predictions of cluster formation and stability under experimental conditions. This guidance in experimental design saves time, reduces costs, and further promotes the development of new materials, new catalysts and new energy technologies. In particular, gold clusters are crucial in Surface-Enhanced Raman Spectroscopy (SERS) and photothermal therapy [21, 22]. However, their structure is exceptionally complex and possesses an abundance of isomers [23–26]. Considering the Au20 cluster [27–29], we display six of its isomer structures, as shown in Fig. 1. Thus, the search for the globally optimal structure among various isomers presents a substantial challenge, and establishing theoretical computational models for cluster energy holds significant research value and promising applications.

The theoretical calculation models of cluster energy can be mainly divided into two categories. One category is the ab initio method, rooted in the first principles of quantum mechanics [30, 31], which includes Density Functional Theory (DFT) [32, 33], Hartree-Focktheory (MF) [34], Second-order Moller–Plesset Perturbation theory [35, 36], Complete Active Space Perturbation theory (CASPT2) [37], Multi-Reference Configuration Interaction (MRCI) [38] and others. These methods predict the energy of clusters by describing the electronic structure and interactions among electrons. Their main challenge lies in analysing wave functions in a high-dimensional space, requiring complex computations with many degrees of freedom and parameters. As the number of atoms increases, so does the computational complexity, exponentially increasing demands on computing resources and time. Therefore, it is not an ideal or efficient solution in practical applications. The other category relies on empirical potential energy function methods, primarily including Lennard–Jones [39, 40], Morse [20, 41], Gupta [42, 43], Sutton-Chen [44, 45] and Reactive empirical bond order (REBO) [46, 47]. These methods provide approximate predictions of cluster energy by constructing empirical potential energy functions to describe atomic interactions. While offering fast computational speed and low computational cost, their precision and applicability are significantly limited by the selected potential energy function and its associated parameters. The rapid advancement of artificial intelligence has prompted researchers to increasingly utilize machine learning techniques for challenges in regression and classification. Machine learning methods, being data-driven in nature, make decisions by discerning patterns and associations within datasets. Consequently, they find widespread application across various domains, including computational physics, chemistry, and materials science. Hansen et al. [48] employed the linear regression method to establish the relationship model between the structure information and energy of clusters, successfully achieving energy prediction. The model demonstrated good performance, and the experiment illustrated that the application of machine learning methods to describe atomic interactions can accelerate the energy prediction process.

However, there are two issues that need to be explored and addressed: (1) The energy prediction model, which relies on traditional machine learning methods, encounters challenges. It depends on human expertise and struggles with slower processing speed and greater computational requirements when dealing with the intricate relationship between cluster structure and energy in high-dimensional nonlinear data. (2) The performance of the model is closely linked to the hyperparameters' values. Previous methods for setting hyperparameters through exhaustive searches were not only less efficient but also produced unsatisfactory results, particularly in scenarios with a large number of hyperparameters.

This paper puts forward feasible solutions for tackling the two aforementioned issues. Firstly, we draw inspiration from several advanced machine learning methods, including Random Forest (RF), Gradient boosting decision tree (GBDT), eXtreme Gradient Boosting (XGBoost), and LightGBM. These methods have demonstrated impressive performance in handling highly nonlinear feature problems [49–54]. Given LightGBM's strong robustness and resilience [55, 56], we propose employing it for cluster energy prediction. Secondly, swarm intelligence optimization algorithms offer notable benefits in the field of optimization. Specifically, the WOA stands out for its fewer parameters, ease of implementation and adjustment, and efficient global search capability [57–61]. Therefore, we utilize the WOA to search for hyperparameters.

The main contributions of this paper are as follows:

1. We employ an advanced machine learning technique to predict the energy of Au20 clusters. By analysing the relationship between atoms, we transform the spatial structure information of the cluster into a numerical matrix and extract its features. By utilizing this feature sequence as input and energy as output, we establish the LightGBM model, introducing a novel approach to predicting cluster energy.
2. Various strategies are proposed to enhance the WOA, including adjustments in population initialization, linear changes in the convergence factor, and a fixed whale weight, resulting in the development of the MSFWOA with improved performance. Applying this algorithm to search for optimal hyperparameters for the LightGBM model offers a fresh idea on parameter optimization.
3. The model exhibits excellent scalability and holds promising application prospects in fields such as materials chemistry, condensed matter physics, and biomedical research.

## Methodology and modeling

### Methodology

#### Feature representation

Choosing the appropriate cluster representation method is crucial for the performance of machine learning methods. For a cluster containing *N* atoms, we require a function $E^N$: $R^{3N} \times N \to R$ to convert the cluster's structural information into a numerical vector. Thus, we adopt the Coulomb representation proposed by Rupp et al. [62]. Based on the atom's nuclear charge and three-dimensional coordinates, the cluster data is encoded into a $N \times N$ dimensional matrix. The equation for the Coulomb matrix is as follows:

1 $$ C_{ij} = \left\{ \begin{gathered} 0.5 \times Z_i^{2.4} ,\;\forall i = j \hfill \\ \frac{Z_i Z_j }{{\left| {R_i - R_j } \right|}},\;\forall i \ne j \hfill \\ \end{gathered} \right. $$

where $Z_i$ and $R_i$ denote the nuclear charge and the three-dimensional spatial coordinates of atom $i$, respectively. The matrix's diagonal elements are derived by fitting the total energy of free atoms and the nuclear charge using a polynomial, while the off-diagonal elements signify the Coulombic repulsion between two atoms within the cluster.

Subsequently, we compute eigenvalues for the $N \times N$ Coulomb matrix to extract its characteristics. Eigenvalues are crucial in representing matrix information and are commonly used for matrix dimensionality reduction. The equation for eigenvalue computation is as follows:

2 $$ \left[ {\begin{array}{*{20}c} {C_{11} } & {C_{12} } & {C_{13} } & \cdots & {C_{1N} } \\ {C_{21} } & {C_{22} } & {C_{23} } & \cdots & {C_{2N} } \\ {C_{31} } & {C_{32} } & {C_{33} } & \cdots & {C_{3N} } \\ \vdots & \vdots & \vdots & \ddots & \vdots \\ {C_{N1} } & {C_{N2} } & {C_{N3} } & \cdots & {C_{NN} } \\ \end{array} } \right]\mathop{\longrightarrow}\limits^{Cx = \lambda x}\left[ {\begin{array}{*{20}c} {\lambda_1 } \\ {\lambda_2 } \\ {\lambda_3 } \\ \vdots \\ {\lambda_N } \\ \end{array} } \right] $$

The eigenvalues obtained from Eq. (2) are used as the feature sequence for the Au20 cluster.

#### Light gradient boosting machine

LightGBM [63] represents a highly efficient distributed ensemble algorithm that evolved from GBDT [64] in 2017. The core idea of GBDT is to substitute the output residuals of the previous tree with the direction of the steepest descent in the loss function (negative gradient direction) to generate a new decision tree. During the iteration, GBDT keeps the current model unchanged and relearns a function to approximate the actual values more accurately. The ultimate prediction results are obtained by combining the outputs of multiple decision trees.

Given $X = \left( {x_1 ,x_2 , \cdots ,x_N } \right)$ and $Y = \left( {y_1 ,y_2 , \cdots ,y_N } \right)$, the model is shown as Eq. (3):

3 $$ F_K \left( X \right) = \sum_{k = 1}^K {f_k \left( X \right)} $$

where $f_k \left( X \right)$ denotes the k-th decision tree, and $K$ is the total number of decision trees.

Initialize the model with $F_0 \left( X \right) = 0$. At the ${\text{t}}$-th iteration, the model and loss function are expressed as Eqs. (4, 5) respectively:

4 $$ F_t \left( X \right) = F_{t - 1} \left( X \right) + f_t \left( X \right) $$

5 $$ L\left( {Y,F_t \left( X \right)} \right) = \sum_{i = 1}^N {L\left( {y_i ,F_t \left( {x_i } \right)} \right)} = \frac{1}{N}\sum_{i = 1}^N {\left( {y_i - F_t \left( {x_i } \right)} \right)^2 } $$

The first derivative $L^{\prime}$ and the second derivative $L^{^{\prime\prime}}$ of the loss function $L$ are calculated by Eqs. (6, 7).

6 $$ L^{\prime}\left( {Y,F_t \left( X \right)} \right) = - \frac{2}{N}\sum_{i = 1}^N {\left( {y_i - F_t \left( {x_i } \right)} \right)} $$

7 $$ L^{\prime \prime}\left( {Y,F_t \left( X \right)} \right) = 2 $$

According to Eq. (4) and the first-order Taylor expansion $f\left( {x + \Delta x} \right) = f\left( x \right) + f^{\prime}\left( x \right) \times \Delta x$, the first derivative $L^{\prime}$ is modified to Eq. (8).

8 $$ L^{\prime}\left( {Y,F_t \left( X \right)} \right) = L^{\prime}\left( {Y,F_{t - 1} \left( X \right) + f_t \left( x \right)} \right) = L^{\prime}\left( {Y,F_{t - 1} \left( X \right)} \right) + L^{\prime \prime}\left( {Y,F_{t - 1} \left( X \right)} \right) \times f_t \left( X \right) $$

$L^{\prime}\left( {Y,F_t \left( X \right)} \right) = 0$, the t-th decision tree is the Eq. (9):

9 $$ f_t \left( X \right) = - \frac{{L^{\prime}\left( {Y,F_{t - 1} \left( X \right)} \right)}}{{L^{\prime \prime}\left( {Y,F_{t - 1} \left( X \right)} \right)}} = - \frac{{ - \frac{2}{N}\sum_{i = 1}^N {\left( {y_i - F_{t - 1} \left( {x_i } \right)} \right)} }}{2}\; = \;\frac{1}{N}\sum_{i = 1}^N {\left( {y_i - F_{t - 1} \left( {x_i } \right)} \right)} $$

Substituting it into Eq. (4), the t-th learner is the Eq. (10):

10 $$ F_t \left( X \right) = F_{t - 1} \left( X \right) + \frac{1}{N}\sum_{i - 1}^N {\left( {y_i - F_{t - 1} \left( {x_i } \right)} \right)} $$

Ultimately, obtaining the strong learner, which represents the optimal solution of the model.

11 $$ F_K (X) = F_0 (X) + \sum_{k = 1}^K {f_k (X)} = F_0 (X) + \sum_{k = 1}^K \frac{1}{N} \sum_{i = 1}^N {(y_i - F_{k - 1} (x_i ))} $$

**Fig. 2:**
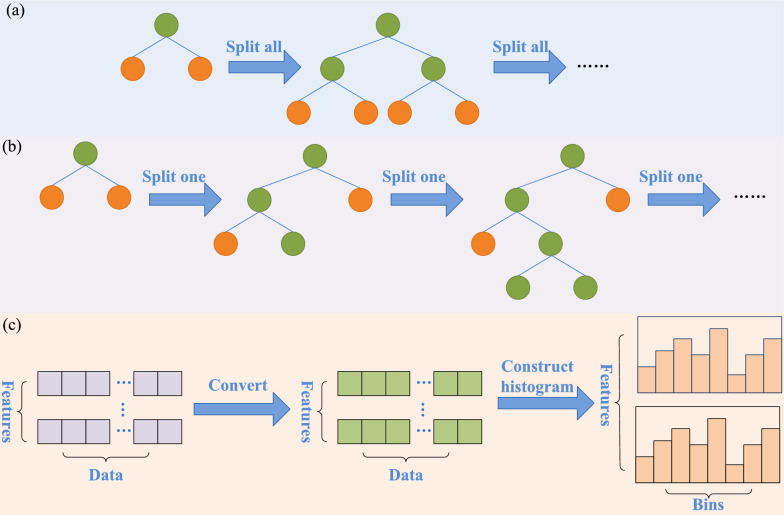
Decision Tree Algorithm Schematic. **a** Level-wise growth strategy; **b** Leaf-wise growth strategy; **c** Histogram-based algorithm

During the construction of the decision tree, GBDT calculates information gain values for all data points and employs a level-wise growth strategy (as shown in Fig. 2a), leading to the challenge of slower model execution speed and higher complexity. LightGBM adopts a histogram-based algorithm (as shown in Fig. 2c), which discretizes continuous features into multiple bins and records information such as the number of samples in each bin. This approach enables the discovery of the optimal split point with just a single pass through the feature data. Additionally, LightGBM employs a leaf-wise growth strategy (as shown in Fig. 2b). This strategy selects only the nodes with the highest gain for splitting through a layer-wise traversal. Consequently, it reduces model complexity and accelerates the training speed.

Moreover, LightGBM utilizes Gradient-based One-Side Sampling (GOSS) and Exclusive Feature Bundling (EFB) for data preprocessing. GOSS retains all high-gradient samples while randomly selecting only a few low-gradient samples, significantly reducing the sample size. After sampling is completed, EFB effectively reduces the feature count by bundling a group of features, which are not exclusively non-zero values, into a new feature package. This process merges features with almost no loss. Furthermore, LightGBM is also optimized for parallel computation. In summary, LightGBM has faster speed than deep neural networks and higher precision than other machine learning methods. As a result, we choose LightGBM for the energy prediction task of the Au20 cluster.

#### Whale optimization algorithm

The WOA, a novel intelligent search algorithm that simulates the hunting behavior of humpback whales, was proposed by Mirjalili et al. [65] in 2016. It is. The algorithm has three phases: encircling prey, bubble-net attacking, and random search. During the whale’s search process, there is a 50% random probability $p$ of choosing to encircle or attack the prey. Meanwhile, the value of parameter $A$ determines whether to expand the predatory search circle or to shrink the encircling circle, changing based on the convergence factor $a$.

12 $$ A{ = }2 \times \left( {r - 1} \right) \times a $$

13 $$ a = 2 \times \left( {1 - \frac{t}{T}} \right) $$

where $A$ is a coefficient vector with values ranging of $[ - \;a,a]$. The value of the convergence factor $a$ linearly decreases from 2 to 0, $r$ is a random number between 0 and 1, $t$ and $T$ represents the current iteration number and the maximum number of iterations, respectively.

1. Encircling prey
  When $P < 0.5$ and $\left| A \right| < 1$, the whale’s position is continuously updated based on the optimal individual whale position calculated by the fitness function, thus encircling the prey.
  14 $$ X_i^{t + 1} = X_{Best} - A^t \times D_{iB}^t $$
  15 $$ D_{iB}^t = \left| {C^t \times X_{Best} - X_i^t } \right| $$
  16 $$ C^t = 2 \times r $$
  where $X_i^t$ and $X_i^{t + 1}$ represent the positions of the $i$-th whale in the t-th and ($t + 1$)-th iterations, respectively. $X_{Best}$ denotes the current optimal whale’s position (i.e., the prey's position). $D_{iB}^t$ is the distance between the $i$-th whale and the prey in the $t$-th iteration, and $C$ is a coefficient vector.
2. Random searching prey
  When $P < 0.5$ and $\left| A \right| \ge 1$, we randomly select one whale from the whale group as a search proxy, then update the positions of other whales based on the search proxy's location.
  17 $$ X_i^{t + 1} = X_{Rand} - A^t \times D_{iR}^t $$
  18 $$ D_{iR}^t = \left| {C^t \times X_{Rand} - X_i^t } \right| $$
  where $X_{rand}$ represents the position of the randomly selected whale. D denotes the distance between the $i$-th whale and the selected one.
3. Bubble-net attacking prey

When $P \ge 0.5$, the whale selects the bubble-net feeding mechanism, a unique predation method of the whale. It moves upward in a spiral path while updating the whale's position and conducting a "bubble net attack" to capture its prey.

19 $$ X_i^{t + 1} = X_{Best} + D^{^{\prime}t} \times e^{bl} \times \cos \left( {2\pi l} \right) $$

20 $$ D^{^{\prime}t} = \left| {X_{Best} - X_i^t } \right| $$

where $D^{^{\prime}t}$ is the distance between the $i$-th whale and the current prey in the $t$-th iteration, $b$ is a constant that defines the shape of the logarithmic spiral, and $l$ is a random number between 0 and 1.

#### Multiple strategy fusion whale optimization algorithm

1. Tent chaotic map initialization
  In the WOA, the initial whale positions are randomly generated, which may result in a non-uniform distribution of the whales and an increased risk of falling into local optima. To address this concern, we introduced the Tent chaotic mapping. The chaotic sequence generated by the Tent mapping exhibits both exploratory and random traits. Consequently, using it for population initialization can lead to a more uniform distribution of initial solutions within the solution space, thereby enhancing the algorithm's exploration capabilities and making it easier to find the global optimum.
  21 $$ Z_{n + 1} = \left\{ \begin{gathered} \frac{Z_n }{u},0 \le Z_n < u \hfill \\ \frac{1 - Z_n }{{1 - u}},u \le Z_n \le 1 \hfill \\ \end{gathered} \right. $$
  where $n$ represents the number of mappings, and $Z_n$ is the value of the $n$-th mapping.
2. Cosine convergence factor
  In the optimization process, changes in the search range of the whale swarm play a crucial role in the algorithm's convergence accuracy and efficiency. As illustrated by Eq. (13), the convergence factor $a$ linearly decreases with an increase in the number of iterations, which may lead to an imbalance in the algorithm's search capabilities during the early and later stages of iteration. Having a value of parameter $A$ can balance the algorithm's ability between global exploration and local optimization. Therefore, we design a cosine convergence factor to dynamically adjust the search range and the value of parameter $A$.
  22 $$ a = \left\{ \begin{gathered} 2 \times \cos \left( {\frac{\pi }{2} \times \frac{t}{T}} \right)^\frac{1}{2} ,a\left( t \right) \le a\left( r \right) \hfill \\ 2 \times \cos \left( {\frac{\pi }{2} \times r} \right)^\frac{1}{2} ,a\left( t \right) > a\left( r \right) \hfill \\ \end{gathered} \right. $$
3. Inertia weight updating strategy
  The weight of whales is a fixed value, which is insufficient to handle the complex nonlinear variations during the optimization process. Therefore, based on the cosine nonlinear variation characteristics of the convergence factor $a$, we introduce the inertia weight factor $w$ to adjust the proportion of the global and local search of the WOA so that the algorithm can quickly converge to the local optimal solution while also having a high probability to jump out of the local optimal and perform global search, which helps to improve the search efficiency and quality of the algorithm.
  23 $$ w = w_{\min } + \left( {w_{\max } - w_{\min } } \right) \times a $$
  where $w_{\min }$ and $w_{\max }$ represent the minimum and maximum values of the weight, which are 0.4 and 0.9. Since $a \in \left[ {0,2} \right]$, the value of weight w is $\left[ {0.4,1.4} \right]$. There's a 3/5 probability for local search and a 2/5 probability for global search.

Substituting the inertia weight factor $w$ into Eqs. (14, 17, 19), we get an updated equation for the whale’s position:

24 $$ X_i^{t + 1} = \left\{ \begin{gathered} w^t \times X_{Best} - A^t \times D_{iB}^t \hfill \\ w^t \times X_{Rand} - A^t \times D_{iR}^t \hfill \\ w^t \times X_j + D_{ij}^t \times e^{bl} \times \cos \left( {2\pi l} \right) \hfill \\ \end{gathered} \right. $$

### Model architecture

**Fig. 3:**
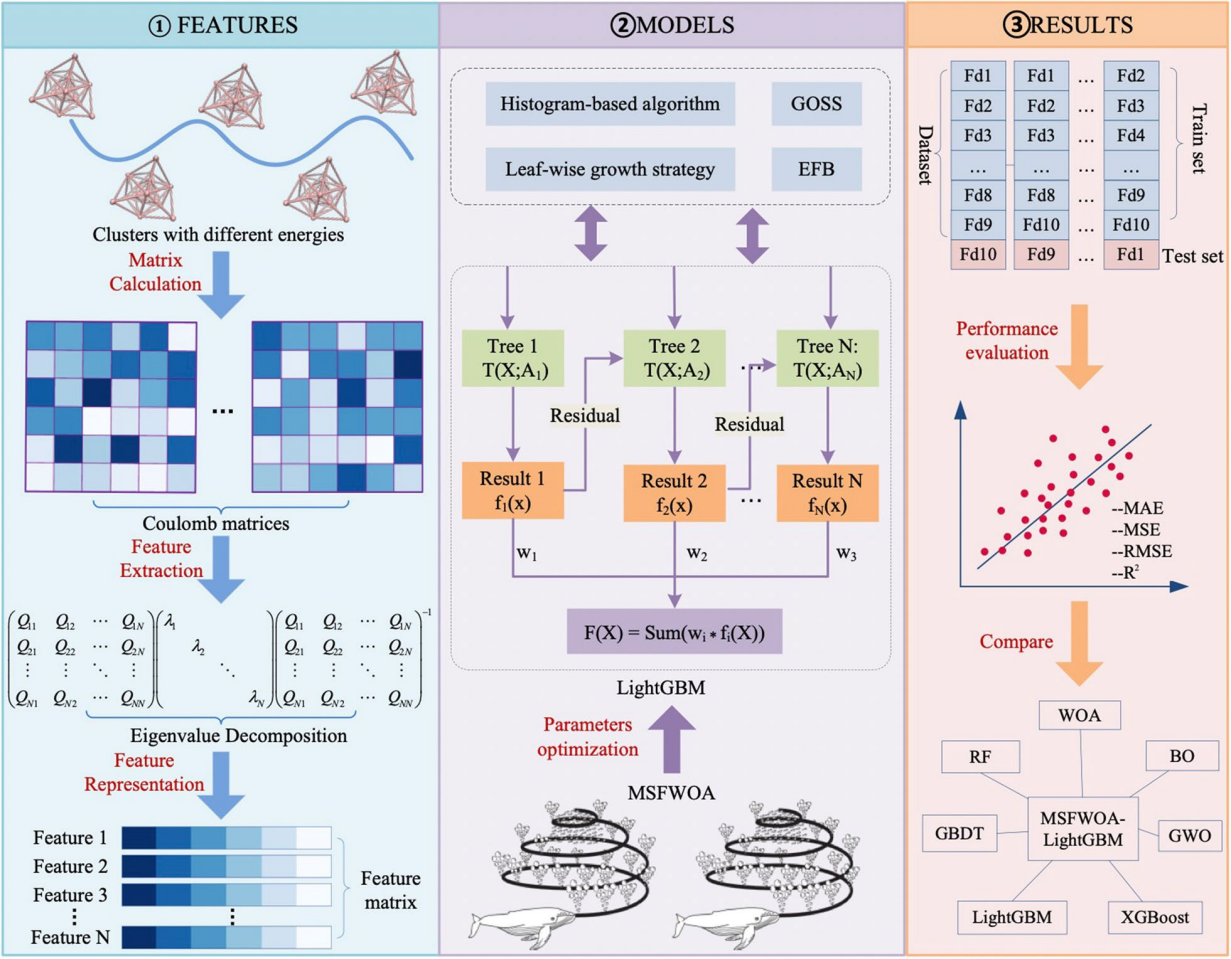
Overview of the method used in this work

The MSFWOA-LightGBM model comprises three stages: feature preparation, model construction, and prediction and analysis, as illustrated in Fig. 3. The first stage is the feature preparation. Using the Coulomb representation, we calculate the atomic coordinates of clusters in the dataset to obtain the Coulomb matrix. Next, we extract features from the Coulomb matrix and compute its eigenvalues to generate a feature sequence. In the second stage, which focuses on model construction, we employed MSFWOA to optimize the LightGBM improved by GBDT, thereby establishing the MSFWOA-LightGBM model. The third stage is the prediction and analysis of the model. We input the feature sequence into the MSFWOA-LightGBM model for training and testing using ten-fold cross-validation, ultimately outputting the energy of Au20. We then conducted a comparative analysis the sample values and the predicted values to assess the model's performance. Meanwhile, we also compare it in multiple aspects with other optimization algorithms and machine learning algorithms to verify the superiority of this model.

### Model evaluation criterion

We adopt Mean Absolute Error (MAE), Mean Square Error (MSE), Root Mean Square Error (RMSE), and Squared Correlation Coefficient (R^2^) to evaluate the effectiveness and accuracy of the proposed model. MAE, MSE and RMSE measure prediction errors, while R^2^ represents the correlation between predicted and actual values, reflecting the model’s fit. The mathematical expressions are as Eq. (25, 26, 27, 28):

25 $$ MAE = \frac{1}{N}\sum_{i = 1}^N {\left| {y_i - \hat{y}_i } \right|} $$

26 $$ MSE = \frac{1}{N}\sum_{i = 1}^N {\left( {y_i - \hat{y}_i } \right)^2 } $$

27 $$ RMSE = \sqrt {{\frac{1}{N}\sum_{i = 1}^N {\left( {y_i - \hat{y}_i } \right)^2 } }} $$

28 $$ R^2 = 1 - \frac{{\sum_{i = 1}^N {\left( {y_i - \hat{y}_i } \right)^2 } }}{{\sum_{i = 1}^N {\left( {y_i - \overline{y}_i } \right)^2 } }} $$

where $N$ represents the number of samples, $y_i$ and $\hat{y}_i$ are experimental and predicted values of the $i$-th sample, respectively, and $\overline{y}$ is the average of all samples.

## Experiments and results

### Data preparation and processing

**Fig. 4:**
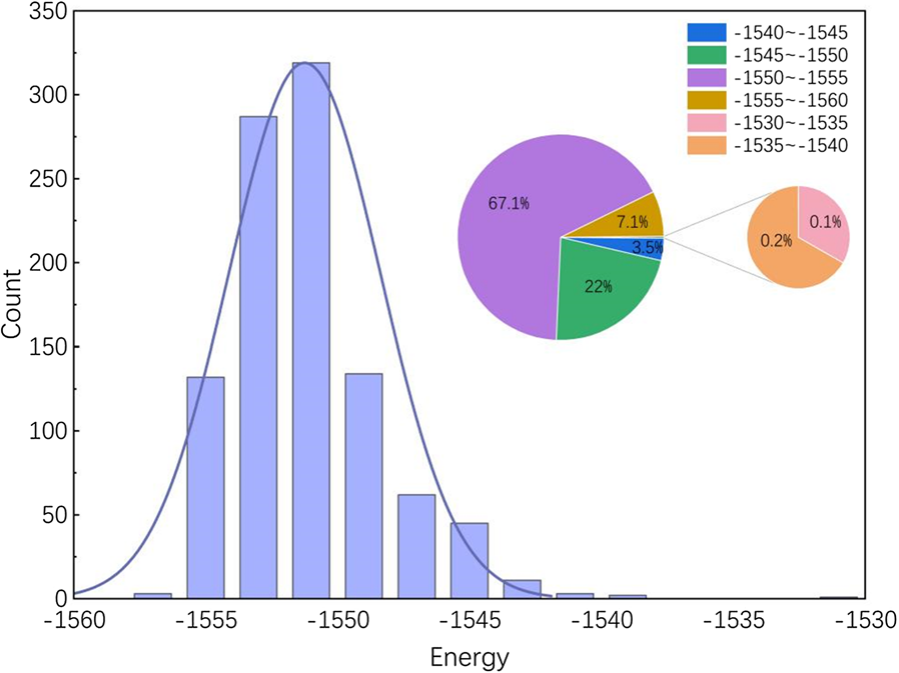
Energy Distribution

The experimental data come from the MathorCup University Mathematical Modeling Challenge, which includes structural files of 999 Au20 clusters. Each file contains a cluster's energy and the three-dimensional Cartesian coordinates of its twenty atoms. By analyzing the dataset statistically, we obtain values for various indicators, such as the maximum value, minimum value, average value, standard deviation, variance, lower quartile, median, and upper quartile, as shown in Table 1. The absolute differences between the upper and lower quartiles and the median for all columns do not exceed 2. The extreme values for the X, Y, and Z axes deviate from the median by less than 10. However, in the energy column, this deviation is 5.553403 from the maximum value and as much as 20.747694 from the minimum value. Figure 4 shows a notably anomalous data point with an energy value of -1530.908363, which deviates significantly from the overall data. Given that this point might influence subsequent research, we consider it an outlier and exclude it from our analysis, focusing solely on the remaining data of 998 gold cluster isomers for further study and analysis.

**Table 1:** The values of the eight statistical indicators about the experimental data

Statistics	X-axis	Y-axis	Z-axis	Energy
Minimum	− 8.44921938	− 8.71675151	− 9.43371008	− 1557.20946
Lower quartile	− 1.378894707	− 1.3220928	− 1.345737223	− 1553.179692
Median	0	0	0	− 1551.656057
Upper quartile	1.37885649	1.32148264	1.378422115	− 1549.891524
Maximum	7.46153601	9.71338212	9.24213362	− 1530.908363
Average	− 0.001298639	0	0.011941082	− 1551.249569
Standard deviation	2.234019222	2.139015713	2.217111666	2.875498361
Variance	4.990841885	4.575388221	4.915584138	8.268490825

### Results

The operating environment is Windows 10 64-bits OS (16 GB of memory and Intel® Core™ i7-8700 processor). The Software is Spyder with python3.7. Initially, based on the nuclear charge and the number of atoms in the Au20 cluster, we calculate the Coulomb matrix and obtain the feature sequence through eigenvalue decomposition. Next, we train and validate the MSFWOA-LightGBM model, using the ten-fold cross-validation and employed MSFWOA to search for the hyperparameter of LightGBM to ensure the model achieves its best performance. Lastly, we evaluate the predictive performance of MSFWOA-LightGBM and analyze the relationship among cluster atom distribution, energy, and structure.

In this experiment, we utilize the MSFWOA algorithm to optimize seven key hyperparameters of LightGBM. The fitness function is the RMSE. During the iterations, we consistently update and track the position of the optimal whale (the prey). Ultimately, we identify a set of hyperparameters that resulted in the lowest value of RMSE, as presented in Table 2. The table presents descriptions, corresponding values, and search ranges for all parameters.

**Table 2:** The hyperparameter values of LightGBM optimized by MSFWOA

Parameter	Description	Value	Range
learning_rate	Boosting learning rate	0.2	(0.01, 0.3)
num_leaves	Maximum tree leaves for base learners	14	(3, 60)
max_depth	Maximum tree depth for base learners	15	(3, 25)
subsample	Subsample ratio of the training instance	0.9	(0.5, 1)
colsample_bytree	Subsample ratio for columns of the tree	0.7	(0.5, 1)
reg_alpha	L1 regularization term on weights	0.5	(0, 100)
reg_lambda	L2 regularization term on weights	3.4	(0, 100)

**Fig. 5:**
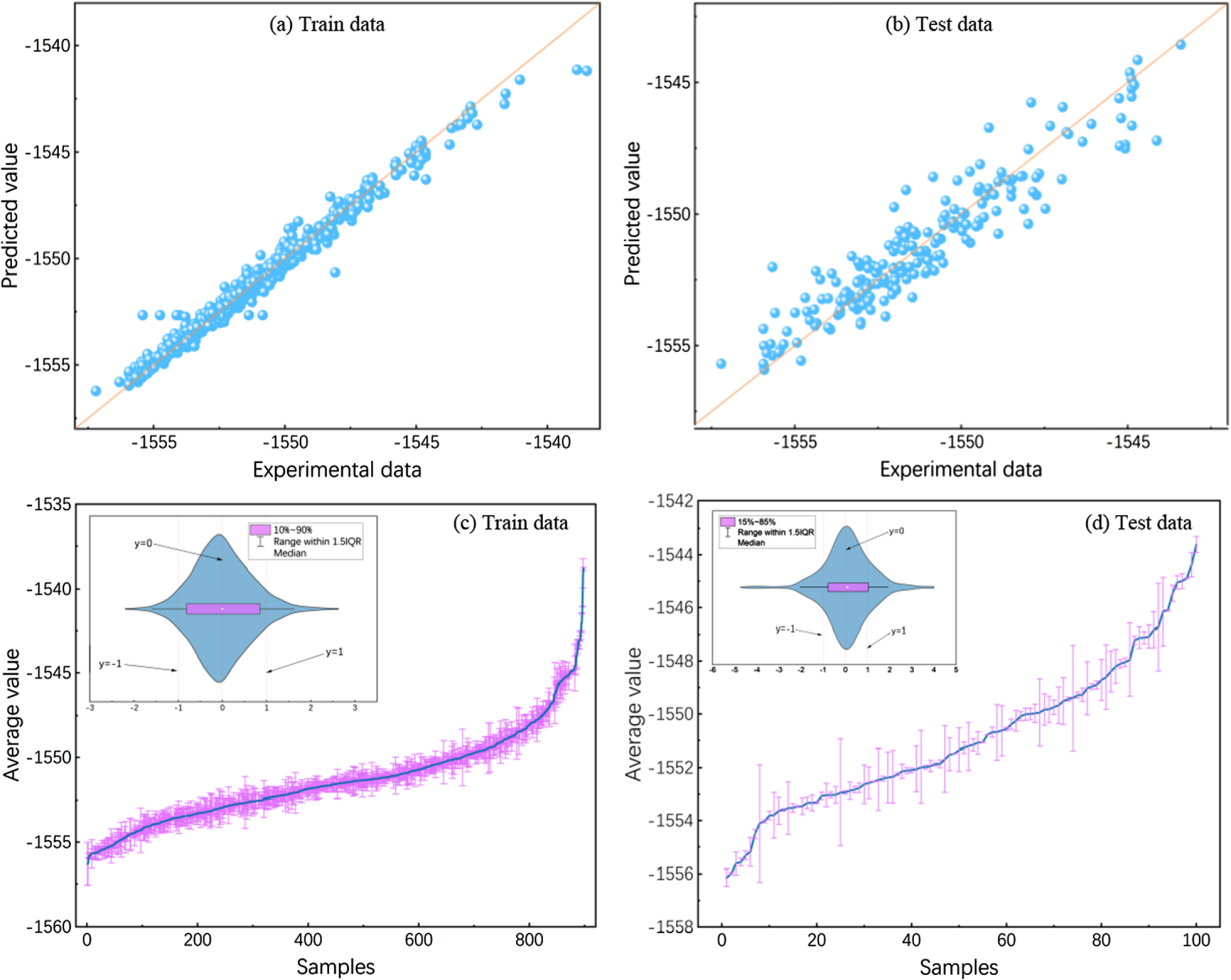
Prediction results and error distribution for the MSFWOA-LightGBM model on the training and test sets. **a** Scatter of predicted and experimental values on the training set; **b** Scatter of predicted and experimental values on the test set; **c **The error distribution for the training set; **d** The error distribution for the test set

According to the experimental results, we compare the difference between the experimental and the predicted value, analyzing the errors, as shown in Fig. 5. The fitting performance of the MSFWOA-LightGBM model for the experimental and predicted values in the training and test sets is illustrated in Fig. 5a, b, respectively. The diagonal indicates that the experimental value is equal to the predicted value. There is a significant count of samples with energy levels ranging from − 1555 to − 1545, whereas the number of samples between − 1545 and − 1540 is limited, displaying a discrete distribution. In Fig. 5a, the data points are all distributed near the diagonal, indicating that the error between predicted and experimental values in the training set is small, demonstrating good model performance. In Fig. 5b, the majority of data points are close to the diagonal, with only a small number of data points in sparsely distributed areas being distant from the line. This observation shows that the non-uniform data distribution has a discernible impact on the model's performance. Overall, the model exhibits a high degree of fitting and performs well.

In Fig. 5c, d, we construct error curve graphs with the average of experimental and predicted results on the vertical axis and used the standard deviation to create error bars. Additionally, we draw violin plots based on error values. In the training set, most error bars ranged from 0 to 1, and only six data points exhibited errors within [1, 2]. In the test set, there are fewer than ten data points with relatively larger error bar lengths, indicating the presence of significant errors. According to the violin plots, 85% of the data had error values within [0, 1], with only three data points having errors greater than 2. Overall, the training and test sets exhibit low errors and few outliers, so the model has high accuracy and good stability.

**Fig. 6:**
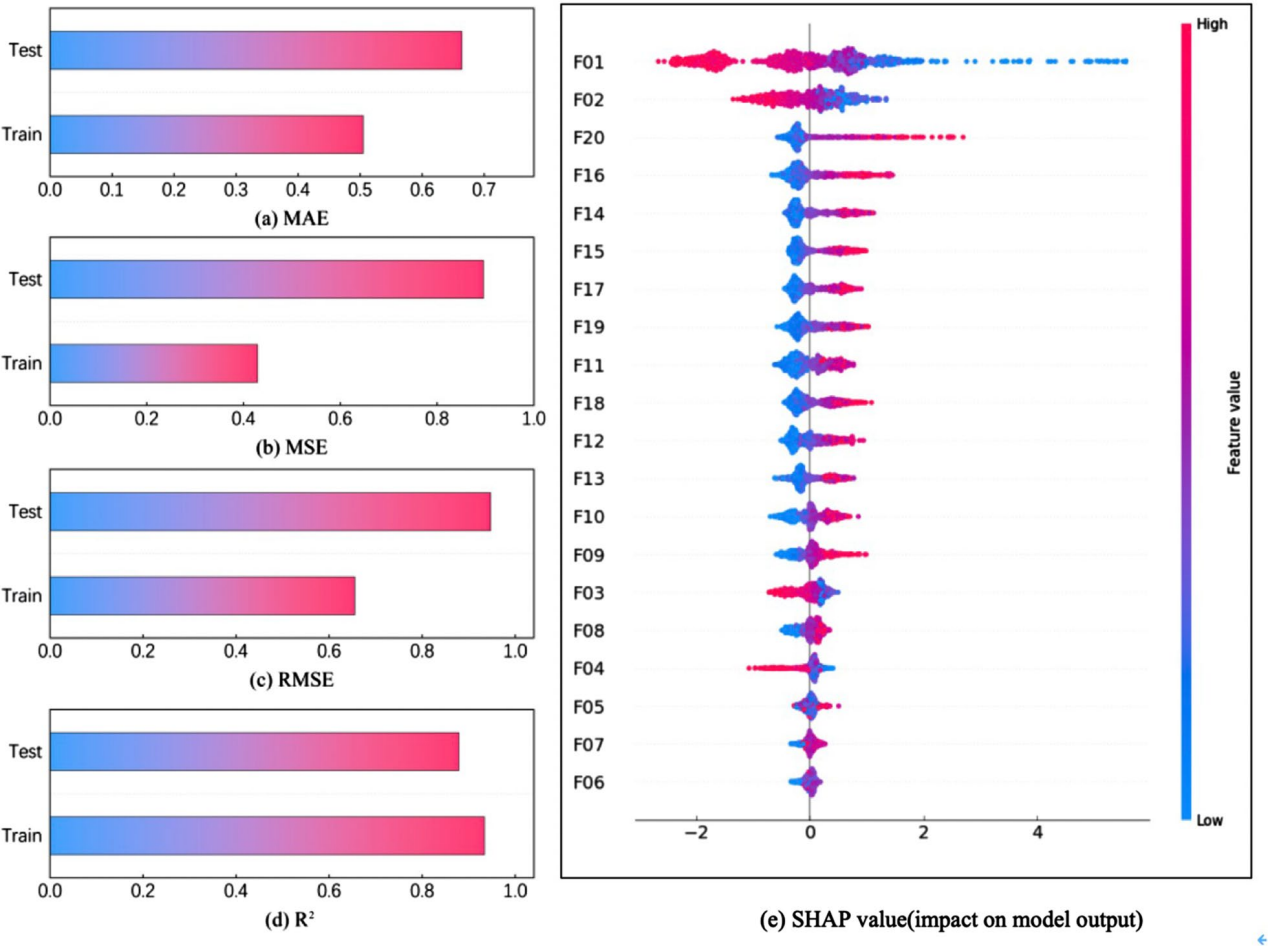
The performance and feature effectiveness of the MSFWOA-LightGBM model. **a** MAE; (**b**) MSE; (**c**) RMSE; (**d**) R^2^; (**e**) SHAP value

To assess the effectiveness of the features extracted by the model, we record the evaluation metric values on the best-performing training and test sets, as well as the values of SHapley Additive exPlanations (SHAP) for the twenty features in this experiment, as shown in Fig. 6. From Fig. 6a–c, the bars in the training set are shorter than those in the test set, with the training set exhibiting smaller MAE, MSE, and RMSE values, indicating lower errors. Additionally, in Fig. 6d, the bars in the training set are slightly taller than those in the test set, and the training set has a higher value of R^2^ compared to the test set, showing a stronger correlation coefficient. Therefore, the model undergoes thorough training and shows excellent generalization performance. As for Fig. 6e, it illustrates the distribution of feature impacts on the model's output. The horizontal axis displays the sum of SHAP’s values, sorted by feature. Each point represents a sample, where the color indicating the feature’s value: red for high values and blue for low values. From the figure, all twenty features impact the model's output, demonstrating their effectiveness.

**Fig. 7:**
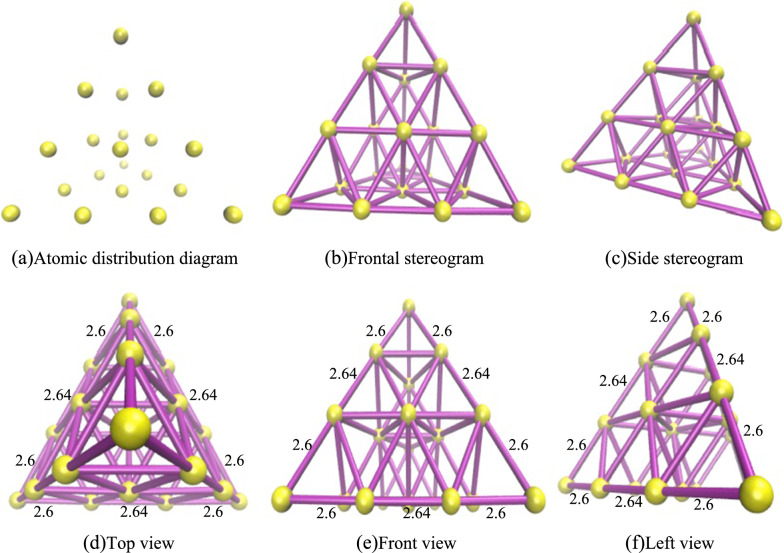
Structure of the Au20 cluster with the lowest energy. **a** Atomic distribution diagram; **b** Frontal stereogram; **c** Side steregram; **d** Top view; **e** Front view; **f** Left view

We utilize the Visual Molecular Dynamics (VMD) software to visualize the Au20 cluster with the lowest energy and find it to be a regular tetrahedral structure, as depicted in Fig. 7. The yellow nodes represent atoms, and the purple segments are connections between adjacent atoms when the Distance Cutoff of DynamicBonds is 2.8. The stereogram consists of twenty atoms and sixty bonds, with each atom bonded to multiple neighboring atoms. Within it, there are four atoms connected to three neighboring atoms, four atoms to nine, and twelve atoms to six, respectively. In three views, it's clear that each face of the tetrahedron is an identical equilateral triangle. Hence, the tetrahedron is a regular tetrahedron, and the atoms on each face are equivalent, exhibiting tetrahedral symmetry. In summary, the structure of the Au20 cluster with the lowest energy primarily exhibits a dense and uniform atomic distribution. The structure is highly symmetrical, rotationally invariant, and tightly packed tetrahedral.

## Analysis and discussion

### Analysis of results with various parameter optimization algorithms

**Fig. 8:**
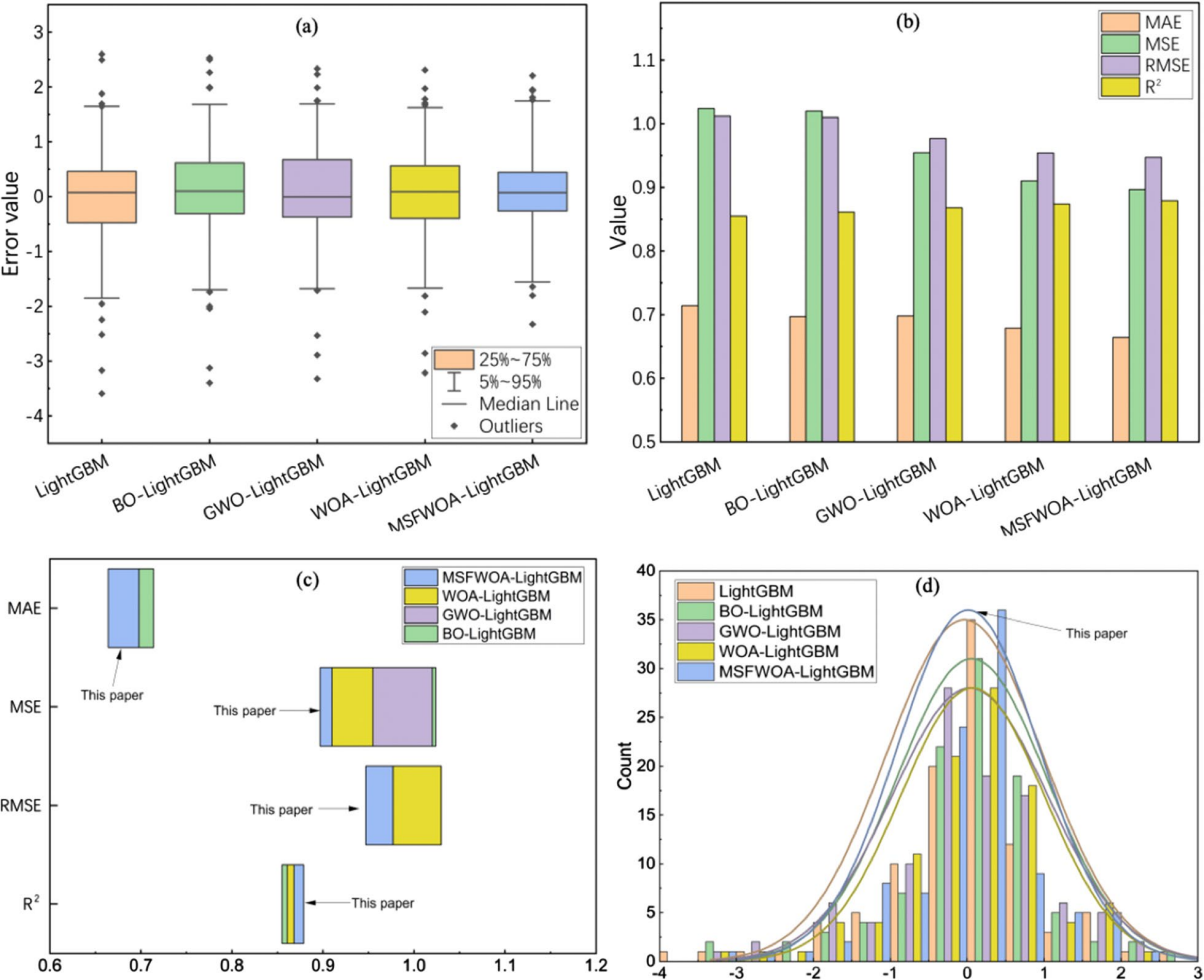
Performance Comparison of Various Parameter Optimization Algorithms. **a** Distribution of errors ; **b** Values of evaluation metrics; **c** Comparison of evaluation metrics; **d** Count distribution of errors

We compared the Bayesian optimization algorithm (BO), the Grey Wolf optimization algorithm (GWO), and the WOA with our proposed MSFWOA algorithm, as referenced from Qiu et al. [66]. To maintain consistency and fairness in the comparison across different optimization algorithms, we utilized a uniform LightGBM parameter search range. Additionally, in order to visually demonstrate the performance differences among the algorithms, we conducted statistical analyses of the errors and various evaluation metrics, as shown in Fig. 8. Figure 8a, d show the Boxplot of sample value errors and the error count statistics for various parameter optimization algorithms on the test set. The rectangular box represents 50% of the data, with the line inside the box indicating the median. The Upper whisker and Lower whisker depict the range of 80% of the data, while the diamond-shaped data points indicate significant outliers. The evaluation metric data for various algorithms on the test dataset is respectively displayed in Fig. 8b, c.

In Fig. 8a, the LightGBM model has more outlier data points than other models, and these outliers exhibit the greatest deviation. This phenomenon indicates that the LightGBM model has a higher number of errors with larger error values. The BO-LightGBM, GWO-LightGBM and WOA-LightGBM models have roughly the same number of outlier data points. However, the WOA-LightGBM model has smaller outlier values, indicating that the LightGBM model, optimized through WOA, exhibits more stable predictive performance. Therefore, WOA demonstrates stronger optimization capabilities than BO and GWO, showing a certain advantage. Furthermore, for the MSFWOA-LightGBM model, the median of the boxplot is the smallest, and the majority of data points are clustered around the median. This phenomenon suggests that this model has the smallest overall error, with most errors falling within the range of [− 3, 3]. In Fig. 8d, it can be observed that MSFWOA-LightGBM has the highest bar near 0, suggesting that the majority of data points exhibit errors centered around 0. From the two bar charts, the values of MAE, MSE, and RMSE for the LightGBM model with parameter optimization algorithm are all smaller than those of the original LightGBM model, while the R^2^ value is greater than that of the original LightGBM model. Furthermore, the MSFWOA-LightGBM model exhibits the smallest value for MAE, MSE and RMSE, as well as the largest value for R^2^. Overall, it is evident that the LightGBM model with parameter optimization algorithm performs more robustly than the one without optimization, and the improved MSFWOA-LightGBM model excels at minimizing prediction errors, delivering superior performance.

### Analysis of results with different machine learning methods

**Fig. 9:**
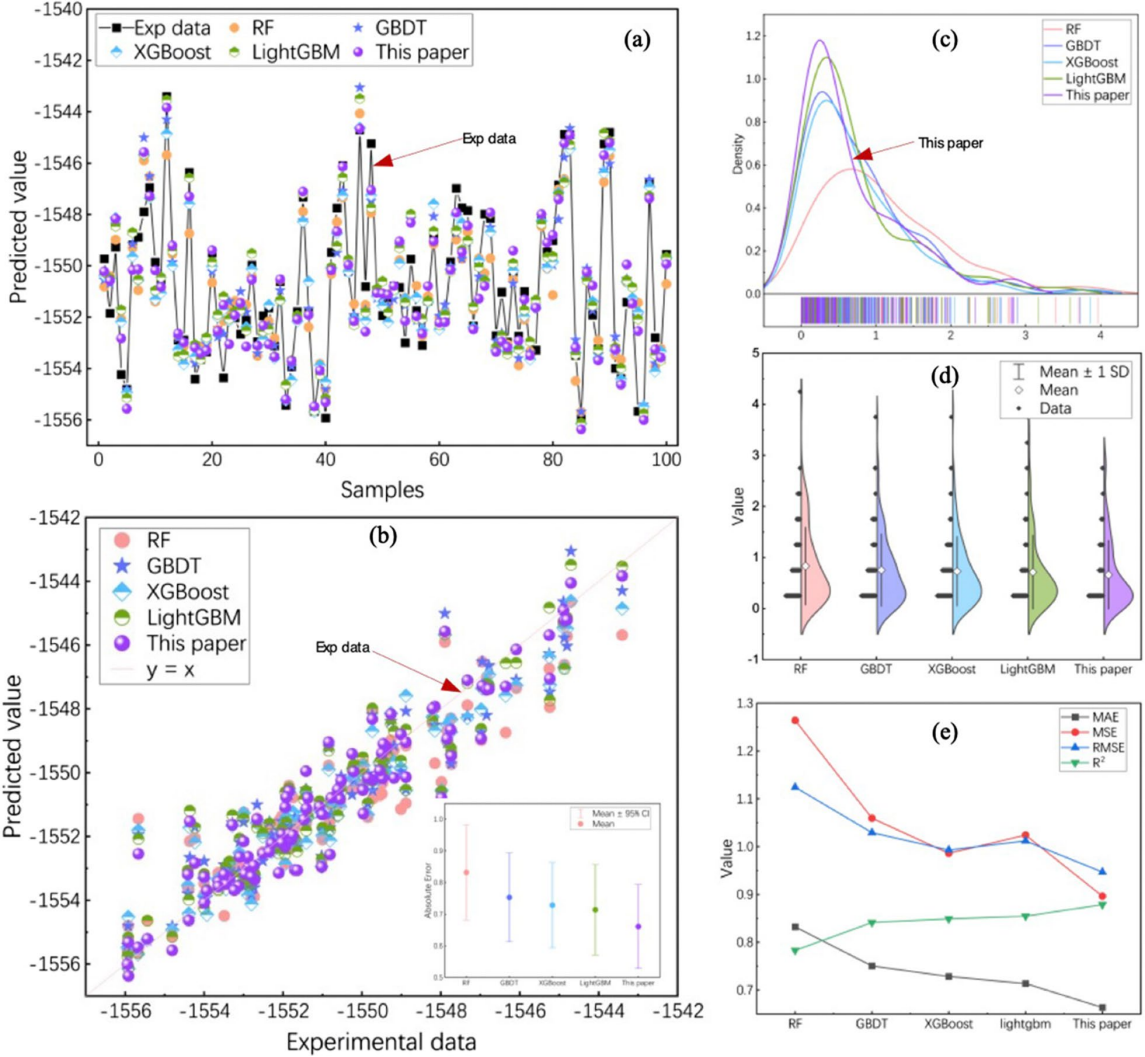
Result and performance of various machine learning models in the test set. **a** Vertical distance distribution of predicted and experimental values; **b** Scatter distribution of predicted and experimental values; **c** Density distribution of absolute errors; **d** Distribution of absolute errors; **e** Line plots of evaluation metrics for different methods

Following the methodologies described by Li et al. [67], we selected four machine learning algorithms, including RF, GBDT, XGBoost and LightGBM, for comparison with the model proposed in this paper. These algorithms were selected based on their proven effectiveness and relevance as extensively detailed in Li et al.'s work. And we created prediction distribution charts and performance comparison charts based on the experimental results from all models on the test dataset, as depicted in Fig. 9.

In Fig. 9a, the variation in experimental data is displayed. Data points are the predictions of five models, and the vertical distance between the data points and the points on the line represents the error between the experimental and the predicted values. And the MSFWOA-LightGBM model exhibits a smaller vertical distance than other models. In Fig. 9b, the diagonal is experimental values equal predicted values and the distribution of predictions for five models around this diagonal is displayed. The predicted values of the MSFWOA-LightGBM model are closely clustered around the diagonal. In the bottom-right corner of Fig. 9b, we can see the error distribution for the five models. The line segments represent the distribution of absolute error values, and the central point represents the average error. The MSFWOA-LightGBM model exhibits the smallest overall average absolute error. As shown in Fig. 9c, the higher the peak of the curve, the greater the distribution of data points at the corresponding position. The peaks of MSFWOA-LightGBM, LightGBM, XGBoost, GBDT, and RF gradually decrease. MSFWOA-LightGBM model corresponds to the smallest error values at the peaks. Combining this with Fig. 9d, it is evident that the MSFWOA-LightGBM model has fewer and smaller outlier error points. Overall, the MSFWOA-LightGBM model exhibits the highest prediction accuracy, a smaller error distribution, and greater stability.

When combining the results from Fig. 9e and Table 3, it becomes evident that the WSFWOA-LightGBM model exhibits a significant advantage in terms of model accuracy and correlation. While GBDT, XGBoost and LightGBM demonstrate similar performance, RF not only performs the worst performance but also requires the longest processing time. In comparison to GBDT and XGBoost, LightGBM has reduced its processing time by almost threefold, even when the differences in model performance are not substantial. Therefore, WSFWOA-LightGBM excels in terms of time efficiency and outperforms LightGBM in overall performance.

**Table 3:** Performance of Various Machine Learning Algorithms

Methods	MAE	MSE	RMSE	R^2^	Time (s)
RF	0.832	1.263	1.124	0.783	4.047
GBDT	0.751	1.059	1.029	0.841	1.941
XGBoost	0.728	0.986	0.993	0.849	1.425
LightGBM	0.714	1.024	1.012	0.855	0.445
MSFWOA-LightGBM	0.664	0.897	0.947	0.879	0.345

### Analysis of cluster’s energy and structure

**Fig. 10:**
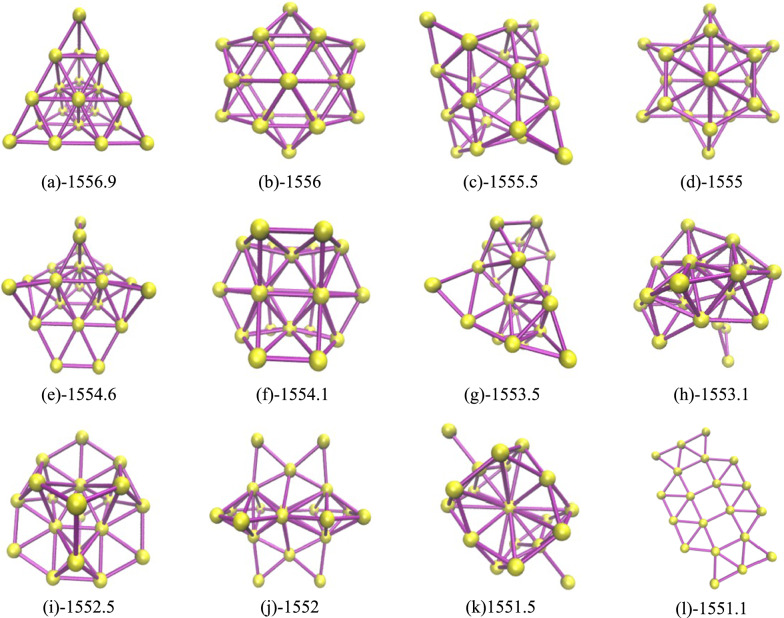
Twelve isomers of the Au20 cluster. **a**–**l** represent the corresponding isomer’s energy value. **a**-1556.9; **b**-1556; **c**-1555.5; **d**-1555; **e**-1554.6; **f**-1554.1; **g**-1533.5; **h**-1553.1; **i**-1552.5; **j**-1552; **k**-1551.5; **l**-1551.1

By comparing different isomers of Au20 clusters, we analyze the relationship between atomic distribution, energy, and structure. The twelve isomers of the Au20 cluster, as shown in Fig. 10, are arranged in ascending order of energy with an energy difference of approximately 0.5 between them, and each isomer exhibits a certain degree of symmetry. In Fig. 10a, a regular tetrahedral structure is evident, consisting of four faces, four vertices, and six edges. The distribution of atoms in space is uniform, demonstrating significant rotational symmetry. It can rotate around four different axes. For three of these axes, each pass through two diagonally opposite vertices. When rotated 180 degrees around these axes, any two vertices coincide, and the entire tetrahedral structure remains unchanged. Another axis is perpendicular to one face of the tetrahedron and passes through the centroid of that face. When rotated 120 degrees around this axis, three vertices coincide in space. The regular tetrahedron also has three planes of symmetry, through which it can be divided into two symmetrical parts. Additionally, it possesses inversion symmetry in space. In Fig. 10b, the atomic distribution is relatively uniform. However, the distance between each pair of atoms is greater than that in a regular tetrahedron, with only one rotation operation. In Fig. 10h, three atoms that are relatively distant from the other 17 atoms. The number of planes of symmetry is limited, with only one present. In Fig. 10l, all atoms are distributed on the same plane. The difference in the number of neighboring atoms for each atom is significant, leading to a non-uniform atomic distribution.

The tetrahedral structure is more stable and has lower energy than other isomers, mainly explained by the following four points: (1) High degree of geometric symmetry. The regular tetrahedral structure, due to its high degree of symmetry, ensures a uniform distribution of atomic spacing. Such a configuration results in a uniform electron cloud distribution, reducing its localization and thus establishing a stable electronic environment. Furthermore, this structure minimizes electron repulsion and instability. The uniform atomic spacing and angles further alleviate structural distortion and internal stress, enhancing stability. As a closed configuration, the regular tetrahedron, with fewer surface atoms and a smaller surface area, significantly reduces surface energy and total energy. (2) Multi-center bonding and balanced coordination environment. The tetrahedral structure allows each atom to form strong Au–Au bonds with multiple neighboring atoms, establishing a multi-center bonding system that enhances the structure's overall stability. This uniform coordination environment leads the system to a more harmonious and balanced state, thereby reducing the system's total energy. It generates a more even charge distribution, reducing the repulsion between electrons and associated instability. (3) Closed-shell structure. The regular tetrahedral structure achieves a fully filled electron shell through the closed-shell configuration, eliminating instability caused by unpaired electrons. It harmonizes with the geometric construct of the tetrahedron, together forming a highly stable and low-energy structure. (4) Lower entropy effect. The highly symmetrical structure limits how atoms can arrange, resulting in a reduction in configurational entropy. It is related to other thermodynamic properties of the system, especially as it lowers the free energy, making this structure more stable in various physical and chemical processes.

Overall, the tetrahedral structure of the Au20 cluster exhibits the lowest energy, due to its high degree of geometric symmetry and optimized electronic structure. This configuration fosters strong interatomic bonding and a balanced electron cloud distribution, establishing it as the most stable isomer.

## Conclusions

In this paper, we utilized the Coulomb representation method and eigenvalue solutions for feature extraction. By incorporating Tent chaos mapping, cosine convergence factor, and inertia weight updating strategy to enhance the WOA algorithm, we developed the MSFWOA. Employing the feature sequence as input and energy as output, with MSFWOA as the parameter search method, we constructed the MAFWOA-LightGBM model for predicting the energy of the Au20 cluster. The experiment demonstrates that Au20 clusters with a regular tetrahedral structure exhibit the lowest energy.

In this structure, the atoms are evenly distributed, and each atom forms strong Au–Au bonds with multiple neighboring atoms. This ensures high symmetry and an optimized electronic structure, provides strong interatomic bonds, and contributes to a uniform electron cloud distribution, thus imparting the structure with the highest stability. The MSFWOA-LightGBM model not only demonstrates excellent predictive performance in energy prediction but also outperforms other comparative models in terms of prediction accuracy, correlation, stability, and computational efficiency. It also gives valuable insights into using swarm intelligence optimization algorithms for parameter tuning. Furthermore, it offers helpful guidance for applying clusters in chemistry, condensed matter physics, and new energy materials. While the MSFWOA-LightGBM model has yielded satisfactory results in the experiments, there are still some issues to explore when investigating the intricate relationship between energy and clusters. For instance, we can develop novel feature encoding methods to better capture cluster information. We can also explore the integration of machine learning techniques with potential energy functions to enhance predictive performance. In the future, we will continue to deepen our understanding of the correlation between atomic distribution, energy, and structure to promote the development of innovative functional materials.

## Data Availability

The datasets generated and/or analyzed during the current study are available free of charge at GitHub. (https://github.com/zhouqi12345678/cluster).

## References

[1] Schleder GR, Padilha ACM, Acosta CM, Costa M, Fazzio A. From DFT to machine learning: recent approaches to materials science-a review. J Phys Mater. 2019;2(3): 032001.

[2] Merabtine N, Djenouri D, Zegour DE. Towards energy efficient clustering in wireless sensor networks: a comprehensive review. Ieee Access. 2021;9:92688–705.

[3] Luo XM, Li YK, Dong XY, Zang SQ. Platonic and archimedean solids in discrete metal-containing clusters. Chem Soc Rev. 2023;52(1):383–444. 36533405 10.1039/d2cs00582d

[4] Roman M, Klokishner S. Electric field effects on magnetic and polarizability properties of clusters with two-electron transfer. J Phys Chem A. 2018;122(46):9093–9. 30383964 10.1021/acs.jpca.8b09822

[5] Batista KEA, Soares MD, Quiles MG, Piotrowski MJ, Da Silva JLF. Energy decomposition to access the stability changes induced by CO adsorption on transition-metal 13-atom clusters. J Chem Inf Model. 2021;61(5):2294–301. 33939914 10.1021/acs.jcim.1c00097

[6] Fang LC, Guo XM, Todorovic M, Rinke P, Chen X. Exploring the conformers of an organic molecule on a metal cluster with bayesian optimization. J Chem Inf Model. 2023;63(3):745–52. 36642891 10.1021/acs.jcim.2c01120 PMC9930108

[7] Xi C, Zheng F, Gao GP, Song ZG, Zhang BY, Dong CK, Du XW, Wang LW. Ion solvation free energy calculation based on Ab initio molecular dynamics using a hybrid solvent model. J Chem Theory Comput. 2022;18(11):6878–91. 36253911 10.1021/acs.jctc.1c01298

[8] Rutledge HL, Rittle J, Williamson LM, Xu WQA, Gagnon DM, Tezcan FA. Redox-dependent metastability of the nitrogenase P-cluster. J Am Chem Soc. 2019;141(25):10091–8. 31146522 10.1021/jacs.9b04555 PMC7445746

[9] Chen J, Luo Z, Yao J. Theoretical study of tetrahydrofuran-stabilized Al_13_ superatom cluster. J Phys Chem A. 2016;120(22):3950–7. 27203626 10.1021/acs.jpca.6b02958

[10] Alonso JA, Lopez MJ. Palladium clusters, free and supported on surfaces, and their applications in hydrogen storage. Phys Chem Chem Phys. 2022;24(5):2729–51. 35077528 10.1039/d1cp03524j

[11] Dânoun K, Tabit R, Laghzizil A, Zahouily M. A novel approach for the synthesis of nanostructured Ag_3_PO_4_ from phosphate rock: high catalytic and antibacterial activities. Bmc Chem. 2021;15(1):42. 34193227 10.1186/s13065-021-00767-w PMC8247164

[12] Santana MVS, Silva De FP. novo design and bioactivity prediction of SARS-CoV-2 main protease inhibitors using recurrent neural network-based transfer learning. Bmc Chem. 2021;15(1):8. 33531083 10.1186/s13065-021-00737-2 PMC7852053

[13] Zhao J, Li M, Gao XG. Construction of SnO2 nanoparticle cluster@PANI core-shell microspheres for efficient X-band electromagnetic wave absorption. J Alloy Compd. 2022;915: 165439.

[14] Chin YC, Yang LX, Hsu FT, Hsu CW, Chang TW, Chen HY, Chen LYC, Chia ZC, Hung CH, Su WC, Chiu YC, Huang CC, Liao MY. Iron oxide@chlorophyll clustered nanoparticles eliminate bladder cancer by photodynamic immunotherapy-initiated ferroptosis and immunostimulation. J Nanobiotechnol. 2022;20(1):373. 10.1186/s12951-022-01575-7 PMC9367122 35953837

[15] Lu XY, Tong AM, Luo DA, Jiang F, Wei JD, Huang YC, Jiang Z, Lu Z, Ni YH. Confining single Pt atoms from Pt clusters on multi-armed CdS for enhanced photocatalytic hydrogen evolution. J Mater Chem A. 2022;10(9):4594–600.

[16] Zhang J, Li J, Yang YL, Yang C, Dong YY, Lin KF, Xia DB, Fan RQ. Functionalized rare-earth metal cluster-based materials as additives for enhancing the efficiency of perovskite solar cells. Acs Appl Ener Mater. 2022;5(11):13318–26.

[17] Surber E, Mabbs R, Habteyes T, Sanov A. Photoelectron imaging of hydrated carbon dioxide cluster anions. J Phys Chem A. 2005;109(20):4452–8. 16833780 10.1021/jp050061p

[18] Grimsley HR, Mayhall NJ. New local explorations of the unitary coupled cluster energy landscape. J Chem Theory Comput. 2022;18(12):7350–8. 36375209 10.1021/acs.jctc.2c00751

[19] Zapata-Torres G, Fierro A, Barriga-Gonzalez G, Salgado JC, Celis-Barros C. Revealing monoamine oxidase B catalytic mechanisms by means of the quantum chemical cluster approach. J Chem Inf Model. 2015;55(7):1349–60. 26091526 10.1021/acs.jcim.5b00140

[20] Khatun M, Sarkar P, Panda S, Sherpa LT, Anoop A. Nanoclusters and nanoalloys of group 13 elements (B, Al, and Ga): benchmarking of methods and analysis of their structures and energies. Phys Chem Chem Phys. 2023;25(29):19986–20000. 37461397 10.1039/d2cp05833b

[21] Al-Otaibi JS, Mary YS, Mary YS, Thomas R. Evidence of cluster formation of pyrrole with mixed silver metal clusters, Agx-My (x = 4,5, y = 2/1 and M = Au/Ni/Cu) using DFT/SERS analysis. Comput Theor Chem. 2022;1208: 113569.

[22] Chen JX, Gong MF, Fan YL, Feng J, Han LL, Xin HL, Cao MH, Zhang Q, Zhang D, Lei DY, Yin YD. Collective plasmon coupling in gold nanoparticle clusters for highly efficient photothermal therapy. ACS Nano. 2022;16(1):910–20. 35023718 10.1021/acsnano.1c08485

[23] Cao L, Li CY, Mueller T. The use of cluster expansions to predict the structures and properties of surfaces and nanostructured materials. J Chem Inf Model. 2018;58(12):2401–13. 30223645 10.1021/acs.jcim.8b00413

[24] Mondal K, Banerjee A, Ghanty TK. Structural and chemical properties of subnanometer-sized bimetallic Au<sub>19</sub>Pt cluster. J Phys Chem C. 2014;118(22):11935–45.

[25] Tlahuice-Flores A, Santiago U, Bahena D, Vinogradova E, Conroy CV, Ahuja T, Bach SBH, Ponce A, Wang G, Jose-Yacaman M, Whetten RL. Structure of the thiolated Au_130_ cluster. J Phys Chem A. 2013;117(40):10470–6. 24004091 10.1021/jp406665m

[26] Ren HJ, Chen F, Li XJ, He YP. A new insight of structures, bonding and electronic properties for 6-mercaptopurine and Ag_8_ clusters configurations: a theoretical perspective. Bmc Chem. 2019;13:55. 31384803 10.1186/s13065-019-0573-z PMC6661816

[27] Patty JB, Havenridge S, Tietje-Mckinney D, Siegler MA, Singh KK, Hosseini RH, Ghabin M, Aikens CM, Das A. Crystal structure and optical properties of a chiral mixed thiolate/stibine-protected Au-18 cluster. J Am Chem Soc. 2022;144(1):478–84. 34957826 10.1021/jacs.1c10778

[28] Chen S, Xiong L, Wang SX, Ma ZY, Jin S, Sheng HT, Pei Y, Zhu MZ. Total structure determination of Au-21(S-Adm)(15) and geometrical/electronic structure evolution of thiolated gold nanoclusters. J Am Chem Soc. 2016;138(34):10754–7. 27552520 10.1021/jacs.6b06004

[29] Mondal K, Agrawal S, Manna D, Banerjee A, Ghanty TK. Effect of hydrogen atom doping on the structure and electronic properties of 20-atom gold cluster. J Phys Chem C. 2016;120(33):18588–94.

[30] Saikia N, Seel M, Pandey R. Stability and electronic properties of 2D nanomaterials conjugated with pyrazinamide chemotherapeutic: a first-principles cluster study. J Phys Chem C. 2016;120(36):20323–32.

[31] Karttunen AJ, Rowley RL, Pakkanen TA. Ab initio study on adsorption of hydrated Na+ and Cu+ cations on the Cu(111) surface. J Phys Chem B. 2005;109(50):23983–92. 16375388 10.1021/jp054295k

[32] Murcia-Galán RA, Durán SM, Leal-Pinto SM, Roa-Cordero MV, Vargas JD, Herrera LV, Muñoz-Castro A, MacLeod-Carey D, Naranjo TW, Rodríguez-Kessler PL, Hurtado JJ. Antifungal activity of Co(II) and Cu(II) complexes containing 1,3-bis(benzotriazol-1-yl)-propan-2-ol on the growth and virulence traits of fluconazole-resistant <i>Candida</i> species: synthesis, DFT calculations, and biological activity. Bmc Chem. 2023;17(1):135. 37817173 10.1186/s13065-023-01037-7 PMC10563319

[33] Marinescu M, Cinteza LO, Marton GI, Chifiriuc MC, Popa M, Stanculescu I, Zlaru CM, Stavarache CE. Synthesis, density functional theory study and in vitro antimicrobial evaluation of new benzimidazole Mannich bases. Bmc Chem. 2020;14(1):45. 32724899 10.1186/s13065-020-00697-z PMC7382033

[34] Nguyen ALP, Izgorodina EI. Behavior of counterpoise correction in many-body molecular clusters of organic compounds: Hartree-Fock interaction energy perspective. J Comput Chem. 2022;43(8):568–76. 35137436 10.1002/jcc.26814 PMC9303541

[35] Bintrim SJ, Berkelbach TC, Ye H-Z. Integral-direct Hartree-Fock and M{\o}ller-plesset perturbation theory for periodic systems with density fitting: application to the benzene crystal. Arxiv. 2022;18(9):5374–81. 10.1021/acs.jctc.2c00640 35969856

[36] Greenwell C, Rezac J, Beran GJO. Spin-component-scaled and dispersion-corrected second-order Moller-Plesset perturbation theory: a path toward chemical accuracy. Phys Chem Chem Phys. 2022;24(6):3695–712. 35080535 10.1039/d1cp04922d

[37] Hu QH, Johannesen AM, Graham DS, Goodpaster JD. Neural network potentials for reactive chemistry: CASPT2 quality potential energy surfaces for bond breaking. Digital Discov. 2023;2(4):1058–69.

[38] Goncalves CEM, Galvao BRL, Braga JP. Accurate multi-reference study of Si_3_ electronic manifold. Theoret Chem Acc. 2016;135(5):116.

[39] John C, Swathi RS. Global optimization of dinitrogen clusters bound to monolayer and bilayer graphene: a swarm intelligence approach. J Phys Chem A. 2023;127(21):4632–42. 37195030 10.1021/acs.jpca.3c01399

[40] Iida Y, Hiratsuka T, Miyahara MT, Watanabe S. Mechanism of nucleation pathway selection in binary lennard-jones solution: a combined study of molecular dynamics simulation and free energy analysis. J Phys Chem B. 2023;127(15):3524–33. 37027488 10.1021/acs.jpcb.2c08893

[41] Hou D, Zhai Y, Sun TT, Zhang XL, Li H. Vibrationally excited intermolecular potential energy surfaces and the predicted near infrared overtone (v(OH) = 2 <– 0) spectra of a H(2)O-Ne complex. Phys Chem Chem Phys. 2022;24(21):12937–49. 35604277 10.1039/d2cp01407f

[42] Guleria K, Verma AK, Goyal N, Sharma AK, Benslimane A, Singh A. An enhanced energy proficient clustering (EEPC) algorithm for relay selection in heterogeneous WSNs. Ad Hoc Netw. 2021;116: 102473.

[43] Garip AK, Gocen T. The local atomic pressures in 79 atom Pd-Ag-Pt truncated octahedron structure. Eur Phys J Appl Phys. 2022;97:30.

[44] Yang GM, Fan XF, Shi S, Huang HH, Zheng WT. Stability of Pt-n cluster on free/defective graphene: a first-principles study. Appl Surf Sci. 2017;392:936–41.

[45] Zhokh A, Strizhak P, Goryuk M, Narivskiy A. Thermodynamic analysis of Al clusters formation over aluminum melt. Phys Scr. 2021;96(12): 125725.

[46] Qamar M, Mrovec M, Lysogorskiy Y, Bochkarev A, Drautz R. Atomic cluster expansion for quantum-accurate large-scale simulations of carbon. J Chem Theory Comput. 2023;19(15):5151–67. 37347981 10.1021/acs.jctc.2c01149

[47] Aghajamali A, Karton A. Correlation between the energetic and thermal properties of C40 fullerene isomers: an accurate machine-learning force field study. Micro Nano Eng. 2022;14: 100105.

[48] Hansen K, Montavon G, Biegler F, Fazli S, Rupp M, Scheffler M, von Lilienfeld OA, Tkatchenko A, Muller KR. Assessment and validation of machine learning methods for predicting molecular atomization energies. J Chem Theory Comput. 2013;9(8):3404–19. 26584096 10.1021/ct400195d

[49] Gupta VK, Gupta A, Kumar D, Sardana A. Prediction of COVID-19 confirmed, death, and cured cases in India using random forest model. Big Data Min Analyt. 2021;4(2):116–23.

[50] Jackins V, Vimal S, Kaliappan M, Lee MY. AI-based smart prediction of clinical disease using random forest classifier and Naive Bayes. J Supercomput. 2021;77(5):5198–219.

[51] Wang RR, Wang LP, Zhang J, He M, Xu JG. XGBoost machine learning algorism performed better than regression models in predicting mortality of moderate-to-severe traumatic brain injury. World Neurosur. 2022;163:E617–22. 10.1016/j.wneu.2022.04.044 35430400

[52] Meng DL, Xu J, Zhao JJ. Analysis and prediction of hand, foot and mouth disease incidence in China using random forest and XGBoost. PLoS ONE. 2021;16(12):E0261629. 34936688 10.1371/journal.pone.0261629 PMC8694472

[53] Shaker B, Yu MS, Song JS, Ahn S, Ryu JY, Oh KS, Na D. LightBBB: computational prediction model of blood-brain-barrier penetration based on LightGBM. Bioinformatics. 2021;37(8):1135–9. 33112379 10.1093/bioinformatics/btaa918

[54] Liu X, Liu TQ, Feng P. Long-term performance prediction framework based on XGBoost decision tree for pultruded FRP composites exposed to water, humidity and alkaline solution. Compos Struct. 2022;284: 115184.

[55] Alabdullah AA, Iqbal M, Zahid M, Khan K, Amin MN, Jalal FE. Prediction of rapid chloride penetration resistance of metakaolin based high strength concrete using light GBM and XGBoost models by incorporating SHAP analysis. Constr Build Mater. 2022;345: 128296.

[56] Yoon HI, Lee H, Yang JS, Choi JH, Jung DH, Park YJ, Park JE, Kim SM, Park SH. Predicting models for plant metabolites based on PLSR, AdaBoost, XGBoost, and LightGBM algorithms using hyperspectral imaging of Brassica juncea. Agri Basel. 2023;13(8):1477.

[57] Zhang R, Liu M, Pan ZH, Yin YF. Network security situation assessment based on improved WOA-SVM. Ieee Access. 2022;10:96273–83.

[58] Rezaie F, Panahi M, Bateni SM, Jun C, Neale CMU, Lee S. Novel hybrid models by coupling support vector regression (SVR) with meta-heuristic algorithms (WOA and GWO) for flood susceptibility mapping. Nat Hazards. 2022;114(2):1247–83.

[59] Samantaray S, Sahoo A. Prediction of suspended sediment concentration using hybrid SVM-WOA approaches. Geocarto Int. 2022;37(19):5609–35.

[60] Wang S, Zhang L, Yin G. Vibration prediction and evaluation system of the pumping station based on ARIMA–ANFIS–WOA hybrid model and D-S evidence theory. Water. 2023;15(14):2656.

[61] Liu YF, Yan CP, Ni HX. The approach to multi-objective optimization for process parameters of dry hobbing under carbon quota policy. Int J Adv Manuf Technol. 2022;121(9–10):6073–94.

[62] Rupp M, Tkatchenko A, Müller K-R, Anatole von Lilienfeld O. Fast and accurate modeling of molecular atomization energies with machine learning. Phys Revi Lett. 2012;108(5):058301. 10.1103/PhysRevLett.108.058301 22400967

[63] Ke G, Meng Q, Finley T, Wang T, Chen W, Ma W, Ye Q, Liu TY. Lightgbm: a highly efficient gradient boosting decision tree. Adv Neural Informat Process Syst. 2017;30:3146–54.

[64] Friedman JH. Greedy function approximation: a gradient boosting machine. Annals Stat. 2001;29:1189–232.

[65] Mirjalili S, Lewis A. The whale optimization algorithm. Adv Eng Softw. 2016;95:51–67.

[66] Qiu Y, Zhou J, Khandelwal M, Yang H, Yang P, Li C. Performance evaluation of hybrid WOA-XGBoost, GWO-XGBoost and BO-XGBoost models to predict blast-induced ground vibration. Eng Comput. 2022;38(SUPPL 5):4145–62.

[67] Li M, Chen H, Zhang H, Zeng M, Chen B, Guan L. Prediction of the aqueous solubility of compounds based on light gradient boosting machines with molecular fingerprints and the cuckoo search algorithm. ACS Omega. 2022;7(46):42027–35. 36440111 10.1021/acsomega.2c03885 PMC9685740

